# Effect of Patent Expiry on the Performance of Innovator Multinational Pharmaceutical Companies in a Low Middle Income Country

**DOI:** 10.3389/fmedt.2022.783460

**Published:** 2022-04-04

**Authors:** Farrukh Khalil, Joseph Odhiambo Onyango

**Affiliations:** ^1^Getz Pharma (Kenya), Nairobi, Kenya; ^2^Strathmore Business School, Institute of Healthcare Management, Strathmore University, Nairobi, Kenya; ^3^Strathmore Business School, Strathmore University, Nairobi, Kenya

**Keywords:** patent expiry, loss of exclusivity, performance, innovator multinationals, Low-Middle-Income County, Kenya

## Abstract

Patent expiry or loss of exclusivity exposes innovator pharmaceutical companies to changes in the market dynamics brought about by increased production of generics by rival companies after patent expiration. This current study focused on the effect of generic products manufacturing and competitive market pressures, price changes, and changes in sales volumes and profitability of innovator multinational pharmaceutical companies after patent expiry. The methodology of this study involved a descriptive survey design and utilized both qualitative and quantitative techniques for data collection, analysis, and presentation. Primary data were collected using the key informants' in-depth interviews and survey questionnaires. The top management, including regional managers, general managers, and directors of each of the eight companies participating in this study, were interviewed to gather the qualitative data. Thirty-six respondents comprising of Product Development Managers and Business Supervising Managers responded to a survey questionnaire through purposive sampling. Findings depicted a significant effect of patent expiry on the generic production and subsequent decline in the performance of multinational innovator companies in the pharmaceutical industry. This study recommends that multinational innovator companies operating in low-income countries, such as Kenya, develop strategic policies to tap into the market by leveraging generic production through collaborative manufacturing with generic companies to share revenues.

## Introduction

Patents across various industries, including drugs, arise from the intellectual property spurred from the innovation to raise the rewards for discovery, which also grants a monopoly in discovery. Patents foster innovation as they provide the manufacturer with the opportunity for a temporary monopoly for a period of market exclusivity (1). Consequently, the expiry of patents decreases the overall market returns of individual pharmaceuticals due to post-expiration reduction in marketing share. Loss of patent leads to patent cliff. The patent cliff is a colloquialism exclusively used in the pharmaceutical industry to denote the potential sharp decline in revenues upon patent expiry of one or more leading products of a firm (2, 3).

The patent cliff facing the industry is a reality, with billions of dollars being stripped from companies' revenues (4). The expiration of patents, a concept referred to as the patent cliff, affects multinational pharmaceutical companies with rival companies' production of generic products. The patent cliff is a phenomenon that began in 2010, denoting the issue around patent expiry periods and a sudden dip in the sales margins that come later for the essential products that captivate the high percentage of a market (5). Examples of patent cliffs include Lipitor, an anti-cholesterol blockbuster that is manufactured by Pfizer whose patent expired in 2011 and Plavix, a bestselling anti-coagulant whose patent expired in 2012.

Systematic literature search performed in PubMed on patent expiration's impact on drug prices is evident in published literature depicted from high-income countries (6). Other companies seek approval to develop generic versions of the same drug selling it at much lower prices than the originator company (7). It enhances competitive market pressure affecting overall sales and turnover of innovator multinational pharmaceutical companies. However, in the European Union, governments have developed policies to regulate rewarding potentially innovative drugs on a pricing and reimbursement scheme (8).

Under normal circumstances, blockbuster drugs result in a sale turnover reduction of up to 80% off-patent. This is attributed to other companies seeking approval to develop generic versions of the same drug selling it at much lower prices than the originator company (7). Pharmaceutical companies spend billions of dollars in Research and Development to develop new drugs and other pharmaceutical products (9). Thus, the patents enjoy their invention by making sales without their products being copied, thus having a competitive advantage in the sector. However, Mohan et al. (10) indicated that it takes 10–15 years on average to fully develop a new dosage of the drug to reach the market. In this regard, a significant time of the patent term is consumed before a product enters the market and realizes returns of the expenses.

Emerging from patent loss is blockbuster, a term closely associated with a prevalent drug that generates annual sales of at least $1 billion for the company that innovated and sold it (2, 11). Typical examples are Vioxx, Lipitor, and Zoloft, among others. Consequently, when a drug's patent expires, the market is flooded with generic drugs after the expiry, negatively impacting sales of the blockbuster drug (2, 3). After the expiry of patent protection, generic manufacturers enter the market with drugs equivalent to the Innovator's drug, but typically at a significantly lower price (12).

Blockbuster patents, in this case, refer to the protection of pharmaceutical products having annual sales of above $1 billion (2). Rapid market entry of generic drugs manufactured and sold at much cheaper rates compared to branded products (7). The result is increased product competition affecting overall sales of branded drugs. In recent years, Big Pharma has already lost billions of dollars on the cheaper generics, and market observers project the trend to continue until 2021 (5). Unfortunately, for Big Pharma, mature markets such as the United States, Japan, and Europe offer limited opportunities for additional market share. Therefore, Low Middle Income countries provide the potential of Big Pharma such as the Sanofi, GlaxoSmithKline, and Bayer to capitalize and embrace the untapped markets. Markets in the developing countries contribute 1.4 billion people among the middle-class population within a decade which will account for over 60% of the global GDP growth (13).

“Generic” refers to a product that does not have a trademark or brand name. An ideal example is “paracetamol,” an inactive substance acting as a medium in many pain killers and is majorly sold as a generic drug without a brand name. The term generic is also utilized in referring to duplicates of original drugs whose patents have already expired. The duplicates may only be different in the brand name, but the drug's chemical composition remains the same as the original drug sold under a brand name or trademark (14). Generally, the manufacture of generics or copies of a drug and subsequent marketing occurs under the chemical component (chemical ingredient) or a unique brand name.

Nevertheless, the generic drug should not infringe on intellectual property rights should not be pirated or counterfeited in a separate domain. It is legally allowed for an inventor to produce generics when the original inventor's patent has expired or under voluntary or compulsory license (15). However, generics that are pirated or counterfeit are illegal to produce.

Innovator companies generate products from scratch and patent them as their sole exclusive innovation (16). Innovations in the pharmaceutical industries involve creating new drugs through research and development, targeting specific diseases following a strict process of trials and approval. The company that comes up with a drug formulation for a disease for the first time is the innovator company. Regarding this study, the term innovator refers to pharmaceutical companies' manufacturing original branded products in the industry and possesses patent or rights of exclusivity for their innovation over 20 years (6). The Food and Drug Administration (FDA) approves drug formulations for commercial manufacture and distribution to the target market. The approval for drug manufacture follows stringent measures of evaluation and review before approval to ascertain the veracity of the drug formulation.

The performance involves the capacity of an organization to realize the set goals and objectives in respect to sales, profitability, or growth and development. Generally, an organization is deemed successful if it achieves its goals and objectives effectively while utilizing minimum resources efficiently (17). Thus, many organizations peg their performance to the outcome of profitability. (18) stated that performance involves a combination of financial and non-financial pointers that provides crucial information on the achievement of goals and objectives, as well as results of a given company. From a corporate perspective, performance is viewed as a composite assessment of the execution of organizational activities regarding the financial, market, and shareholder performance. Mbugua (19) asserts that the pharmaceutical sector's performance entails extensive achievement spanning R&D during drug development, increased market penetration, and maintaining a substantive market share to scoop back the used resources.

The performance aspect is dynamic and requires assessment and interpretation to assess and quantify the performance within an organizational set-up. Kariithi and Kihara (20) indicate that manufacturing companies must exemplify capabilities and utilize available resources effectively to achieve superior performance. Good leadership capabilities also contribute to the improved performance of any given organization. From a critical perspective, Peneder (21) states that effective leadership and guidance of employees in the market competitive pressures helps manage the dynamics to ensure the company achieves better returns.

Nigala (22) indicated that pharmaceutical companies ought to ensure a constant flow of production, availability of market, and lack of competitive pressures. Furthermore, organizational performance is affected by the work output of the human capital and thus the need for having the right human resources. Companies put in place competent staff in the production to come up with quality brands and excellent marketers to cut a niche for the brand in the market (23). A competent team aimed at boosting the organization's performance goes beyond following orders to exemplify their skills, competencies, and capacities to improve their work output for organizational excellence. Companies also introduce incentives or compensation of best-performing staff or teams to promote better performance among the staff and overall organizational performance in the sector (24). Thus, overall organizational performance is related to realizing goals and objectives that are set, with profitability and increased sales volumes being key parameters for assessment.

Innovator Multinational Pharmaceutical Companies in Low Middle Income Countries face a significant challenge with their business performance due to patent expiry. Evidence of this had been proved (25) through a secondary analysis of medicine prices, availability, and affordability in 36 developing and middle-income countries. Alternatively, this development has been a concern for the World Health Organization (WHO) as presented in the critical analysis and literature review on local production and access to medicines in low- and middle-income countries (26).

The availability of finances in support of health services at the primary care and critical care affects the growth and development of pharmaceutical companies in Kenya (27). However, Kenya's health sector has failed to achieve the prerequisite of the Abuja Promise of 15% GDP distributions to health care. Indeed, Kenya's total expenditure on health care on average is estimated to be at 4.9% of GDP in 2016 (28). Furthermore, by 2016, Kenya had incurred at least 31 USD per capita on health expenditure of its citizen's services, which mainly was below the expected 34 USD recommendation by the WHO for the African countries to spend on providing a minimal health package to their citizens (28). A recent study on the Kenya health transition report indicates that domestic government expenditures for health have been increasing as a share of total health expenditure in recent years (up from 29% in 2000 to 43% in 2017) (29). WHO ascertains that the Kenyan government has covered about 42.3% of the total expenditures on health, while private expenditure accounts for 59.4% of overall spending. With the lack of enough funding and having a large proportion of the population lacking health insurance, most opt for cheaper drugs or alternatives in Kenya (30). Patent expiry affects the sales of branded products. The expiration of patents brings up the influx of generic products that retail at fair prices and offer better options for the general populace.

The Kenyan pharmaceutical industry consists of 25 multinational pharmaceutical companies involved in the manufacturing and distributing of drugs (31). Kenya is the largest producer and supplier of pharmaceutical products in the COMESA region, contributing to approximately half of the pharmaceutical products market (19). The rapid growth is also evident in locally increased government strategy to improve healthcare access and medication at the county levels. According to the Kenya Pharmaceutical and Healthcare Report 2017, the health sector in the country has a value of $3.5 billion, taking ~6% of the national GDP (32). Currently, there is limited research in Kenya focusing on multinational pharmaceutical companies' response to the effect of patent expiry to shield them from the competition. This study, therefore, aims at answering the question; what is the effect of patent expiry on the performance of multinational pharmaceutical companies in Kenya after patent expiry and come up with recommendations on how to strategically improve the performance of Innovator Multinational Pharmaceutical Companies in Kenya by exploring the following:

1. What is the effect of generic products manufacturing and competitive market pressures after patent expiry on the performance of innovator multinational pharmaceuticals?
2. What effect do price changes have on the performance of innovator multinational pharmaceutical companies after patent expiry?
3. What changes occur in sales volumes and profitability of innovator multinational pharmaceutical companies after patent expiry?

### Scope and Delimitations

This study focused on patent expiry on the performance of innovator Multinational Pharmaceutical Corporations in Kenya. The study findings will help determine the future of pharmaceutical MNCs at the time of patent expiry that will guarantee their competitive advantage and a desirable level of growth of their brands in the face of increasing competitive rivalry. The researcher evaluated various studies on patenting rights and drug development, and outlined a theoretical framework of patent expiry and how it affects the performance of pharmaceutical industries. Diverse studies are looking into factors that affect the performance of pharmaceutical companies in the world to establish strategies to overcome negative market dynamics. However, no concrete studies are looking into patent expiry and its effect in LMICs which form a larger proportion of the target market for pharmaceutical products. Therefore, this study investigates the Kenyan context and aims to assess the impact of patent expiry on the performance of innovator multinational pharmaceutical companies.

### Theoretical Foundations

The study relied on four theories explaining organizational performance and how the loss of exclusivity (LOE) or patent expiration affects the performance of innovator pharmaceutical industries. The theories underpinning this study are Systems Theory, Prospect Theory of patent, Monopoly Pricing theory, and Porter's Five forces. The performance of pharmaceuticals is directly affected by the nature of the industry, hence the application of system theory, policies, and strategies as for patent expiry and pricing aspects

The System Theory is adopted in this study to illustrate the understanding of various aspects within an organizational setting and how they relate. Ng et al. (33) outlined a system as an entity that is a harmonious whole so that a boundary exists around it that distinguishes the internal and external elements. Furthermore, von Bertalanffy (34) defined the system as a composite of various components that interact. A fundamental concept in system theory focuses on the entities' interaction within the whole system.

The concept of the system directly relates to the innovator pharmaceutical companies in a closed system during the rights of exclusivity. The aspect of system theory focuses on the arrangement of an organization and the relations between the various parts connecting it into a holistic entity that is the industry (35). A pharmaceutical company has different operating parts that impact the industry's overall performance depicted in the outcome of returns or profitability. The central aspect in the relationships within innovator pharmaceutical companies is a single autonomous organization's behavior when the production process is impacted by external factors (36). Patent expiration exposes the innovator pharmaceutical companies to the open system phenomenon characterized by the realities of competitive market pressures where generics are imminent and price control is not within the company's advantage.

Edmund Kitch's prospect theory of patents remains critical in understanding and integrating intellectual property with property rights theory. The theory proposes that the utility of intellectual rights of a given company arises after an initial invention giving it the capacity to exploit the opportunity fully (37). The Prospect theory predicts the end of the rivalry with patent rights as a specific company acquires exclusive rights for producing their innovation for a specified time. However, Achuora et al. (36) asserted that the concept of prospecting fails to critically investigate the role of rivalry in a patent system which is a crucial proponent in the performance of manufacturing organizations. The granting of intellectual property rights to pharmaceutical companies offers the opportunity to exploit their innovation for 20 years and employ efficient management strategies to achieve better profitability returns. According to Peneder (21), the patent provides the capacity to commercialize and improve patented technology to acquire advantage of the product in the market without fear of competition. However, the expiry of exclusive rights brings about increased competitive pressure from rival products and substitutes (38).

Monopoly pricing theory involves determining the price of a product by the producer or seller (24). According to (39), patent utilization gives pharmaceutical companies exclusive rights to exploit their research and development and invention in the market. The exclusive rights introduced for 20 years led to legal monopolies by innovative multinational companies, allowing them to set high prices. On the contrary, a manufacturing firm in a perfectly competitive environment is keen on adhering to the price of a good from production to sale to remain competitive with its rivals (40).

Boldrin and Levine (41) indicated that patents for pharmaceutical companies mean, having exclusive rights to produce a drug and follow a similar pricing model of a monopolistic firm. Thus, Innovator multinational pharmaceutical companies can sell low quality goods at higher prices and get good returns. Exclusive rights from a patent ensure that no similar product can be manufactured within the stipulated time, giving the innovator company time to recover its investment (38). However, patent expiry changes the dynamics of the company operations introducing competitive pressures in the market, especially where the barriers to entry are sufficiently low. According to Mele et al. (35), the pharmaceutical industry is not solely characterized by monopolistic production during latent periods.

One of the main aims of every company is to achieve a competitive advantage while striving to create and sustain superior performance. For (42), competition is at the core of the success or failure of firms. As such, the loss of patent becomes a critical element for competitive strategy in the search for a favorable competitive position in the industry beyond patent expiry. In the pharmaceutical industry (43), asserted with a selective survey on theoretical literature on some reflections on the relation between competition and innovation. Porter's five forces best explain the impact of market structure on innovation. The core concepts of Porter's four forces are as follows: a threat by potential entrants, bargaining powers of the suppliers, the threat of the substitute products, and the bargain powers of the buyers. All these forces boil down to industry competition, driving rivalry among firms. About this study, a performance component is attributed to market competition. Porter's generic competitive analysis framework has alluded to this kind of competitive trend. This shows how an organization can sustain a competitive advantage across its chosen market scope to enhance its performance by outdoing its competitors. Empirical evidence from the same market by (44) analyzed competitive, regulatory affairs framework in the Kenyan Pharmaceutical sector using three of Michael Porter's forces for competence powers. This study showed that the most influential buyer group is the multinational headquarters and the weakest is the local marketing department.

The sustainment of the pharmaceutical industry is based on its focus on research and development, making it a highly competitive industry (45). A joint proposition in strategic management literature is the desire to co-align the strategy with the environment based on Porter's five forces (46). However, pharmaceutical products themselves are the main generator of the growth of the world pharmaceutical industry. In this care, therefore, from the perspective of industry rivalry, the dynamics of the industry are characterized by the diverse struggle for existing market share based on innovation, intellectual property rights, and patents on the products. On threats for substitutes, generic brand medication is often seen as the primary substitute for products produced by the pharmaceutical industry (47). However, considering the new entrants in the pharmaceutical industry as a threat is very low because of the high costs required to enter the industry associated with research and development (14). The bargaining powers of buyers are also not high since same as those of suppliers as in the pharmaceutical industry; each supplier holds a certain level of control to be a threat (48).

The four theories form a critical framework for study articulation and expound on the study variables concerning the probable study outcomes. The System theory depicts the innovator pharmaceutical companies from closed and open systems, which directly refers to the duration during rights of exclusivity and after patent expiration. There is less competition within the industry during patent as enjoyed within a closed system. However, the expiry of patent or LOE results in the innovator company exposure to pressure in the industry-leading to changes in prices, sales volumes, and profitability. The Prospect theory focuses on the essence of exclusivity rights, which exterminates rivalry in the industry. Given that patenting plays a considerable role in the pharmaceutical industry and the high likelihood that firms will patent inventions (49). Porter's generic competitive strategies (42) play a critical role in helping pharmaceutical companies sustain competitive advantages when patent protection for the drug expires, as generic drugs are usually developed and sold by a competing company (45).

Furthermore, the monopoly pricing theory clearly outlines the state of innovator companies during the patent duration and their freedom to set prices. The companies are in a monopolistic state of exploiting their innovation. However, the LOE results in the ceasing of monopolistic and competitive pricing arising in the market. From the analysis, all three theories expound on the study concepts. However, system theory touches on the three aspects under consideration. After LOE, increased generic production after the loss of patent and competitive prices arise, and sales and volumes changes crop up (50).

### Conceptual Framework

The three theories form a critical framework for study articulation and expound on the study variables concerning defining the probable study outcomes on the performance of Innovator MNCs

**Figure 1:**
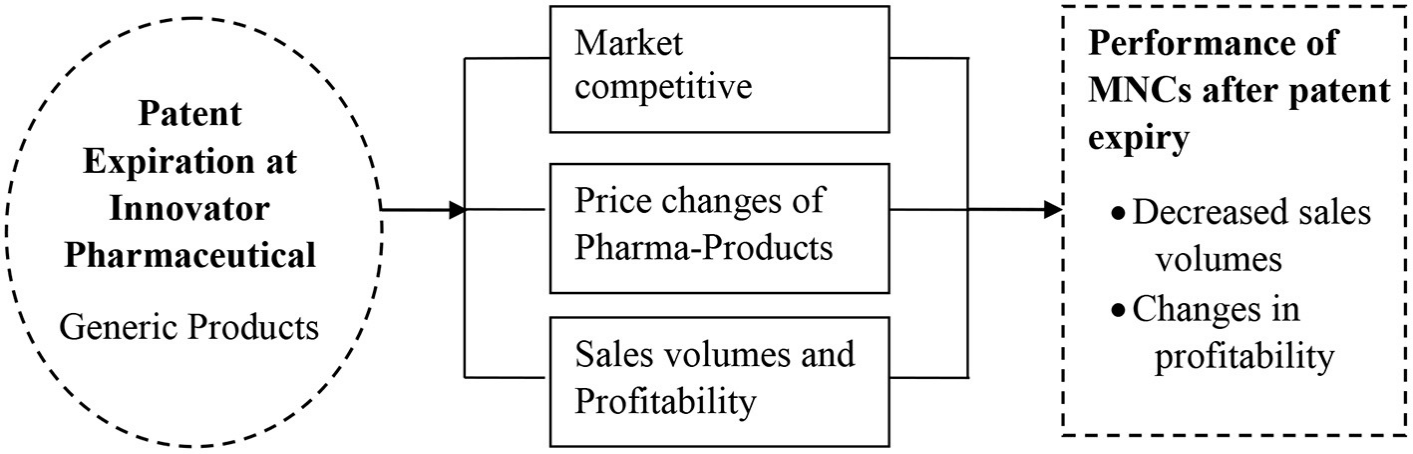
The conceptual framework.

The conceptual framework in Figure 1 presents the aspects of patent expiry of innovator pharmaceutical MNCs affecting their performance and interaction. The effects of patent expiry evaluated are the production of generic products by rival companies, changes in prices, and increased competition in branded products. These are external factors arising and affecting the competitive advantage of pharmaceutical MNCs after the expiry of a patent within the sector. The dependent variable in the study involves the performance of the pharmaceutical MNCs after patent expiry. Addressing the effect of patent expiry ensured a recommendation for continued production, sustained sales, and increased research and development of branded products.

Generic products in the pharmaceutical industry involve drugs processed similarly to the existing approved brand-name drugs and must meet the chemical dosage of the active ingredient, safety measures, quality, strength, administration route, the active ingredients, as well as the mode of performance once administered to users (14). Market competitive forces can break a company. The competition adversely affects the market attractiveness of branded products due to the low costs of generic products (51). In this study, competitive market pressure is created with the rate of generic uptake in the market compared to other branded original products.

Pricing of pharmaceutical products is based on the drug's components, the research and development carried out, and the demand for the product. For example, a medication for a rare disease will cost high compared to the value of a drug for a common condition in society (52). However, the WHO has introduced policies on pricing pharmaceutical products based on countries, a strategy termed cost-plus pricing (28). The significance of price determination of pharmaceutical products varies with geography, risk of the trade, competition of substitutes, and availability of generics (38).

Pharmaceutical industry performance relies on marketing strategies' efficiency and the capacity to penetrate the market to acquire a substantive market share. According to (53), innovator pharmaceutical MNCs acquire exclusive rights to exploit market capacity during the patent period, but the dynamics change drastically after expiration. Factors discussed concerning patent expiry include increased generic production, price changes, and increased competitive pressures. The LOE of a product leads to three options for sustained performance. First, differentiation occurs through product line extension, which increases the product life cycle, route of administration, or new formulations (10).

Market competitive forces can break a company. The competition adversely affects the market attractiveness of branded products due to the low costs of generic products (51). Michael Porter outlined five forces that influence competition within an industry and determine the ultimate profitability within the sector. The first force involves potential entrants in the market and favoring new players in the production process (54). After patent expiration, the entrance of new players becomes probable, with the lapse of exclusivity rights increasing the competitive pressure in the pharmaceutical market of the given drug. However, some barriers bar or hinder new entrants from penetrating such a market and involve a strong brand, economies of scale, significant investment costs, and market experience (55). Thus, innovator pharmaceutical MNCs must embrace strategies that will exploit the barriers of new entrants after patent expiry such as introducing a strong generic product to compete effectively with others.

In this study, competitive market pressure was established by studying the rate of generic uptake in the market compared to branded products. The researcher evaluated the changes in market dynamics by assessing how the introduction of generics after patent expiry affects the demand for branded drugs. This will help the pharmaceutical MNCs focus on the feasibility of sustainability strategies adopted after patent expiry to acquire a competitive edge over the competitors.

The performance of pharmaceutical industries is based on the efficiency of marketing strategies and capacity to penetrate the market to acquire a substantive market share. According to (53), innovator pharmaceutical MNCs acquire exclusive rights to exploit the market capacity during the patent period but upon expiration, the dynamics change drastically. Thus, Nigala (22) states that a multinational company can offer differentiated products by learning more about target customers' needs by adopting the Niche Marketing Strategy. Furthermore, aggressive advertisement promotes the products far and wide to reach more market share. The companies also put in maximum effort to build a strong rapport with their clients to solidify their customer base. Innovator MNCs also utilize a low-cost strategy to compete effectively in the increasingly competitive market to acquire an added advantage over its competitors. Concerning generic production, innovator companies bring out their generics through subsidiaries to tap the need of the customers to buy into the generic products markets due to its affordability.

The LOE of a product leads to three options for sustained performance. First, differentiation occurs through product line extension, which increases the product life cycle, route of administration, or new formulations (10). The second aspect involves reducing incentives for generics and competing through price wars. The ever-present threat of competition from generic product manufacturing requires innovator companies to investigate ways of enhancing their performance strategies. Patent expiration brings about loss of monopoly which the Innovator MNCs can effectively mitigate through market attractiveness and affordable products. Ipert et al. (40) asserted that big pharmaceutical companies have responded to the challenge of reduced performance in the industry by partnerships for production, mergers and acquisition, diversification, consolidation, and reducing the human and capital resources. Thus, the future R&D models should focus on coping with changes in the sector to address cost reduction and delve into areas that bring sustainable growth in the future. The relationship depicted in the conceptual framework demonstrates how the three aspects of generic production, pricing of drugs, and market competitive pressures affect the overall performance of innovator pharmaceutical MNCs. Performance of the companies was assessed through financial and marketing reports analysis pre- and post-patent expiration to give comparative data on the profitability of the products.

## Methods

This study adopted a descriptive research design as the conceptual structural blueprint for data collection, measurement, and analysis to obtain credible discussion information (56). The target population for the research involved 25 pharmaceutical multinational companies in the Kenyan pharmaceutical industry. Through a stratified sampling process, a representative population sample was derived using the concept of at least 30% as depicted by Borg and Gal (57). The study population comprised the eight major multinational pharmaceutical companies in Kenya, representing ~32% of the target population. The companies studied were Pfizer, GSK, Bayer, Sanofi Aventis, Novartis, Astra Zeneca, Eli Lilly, and Roche.

This current study leveraged the primary data collected from the pharmaceutical company that was triangulated with quantitative secondary data collected for the analysis of the results. Thus, the researcher sampled 40 senior supervising managers and supervisors from a total of 283 from the selected companies representing for data collection based on the concept of attaining between 10 and 30% representations.

The researcher embraced a mixed method of data collection which incorporated both quantitative and qualitative data collection methods. The study utilized a survey questionnaire and in-depth interviews to acquire primary data. Secondary data from journals and credible reports were reviewed to support the primary data obtained from the survey. Target secondary data were financial performance and marketing and sales indicators after patent expiry for at least 1-year post-patent expiration (LOEs) to indicate performance changes. The respondents in the study are presented in Table 2 who provided primary data used for analysis.

Qualitative data was gathered through key informant in-depth interviews. One top manager as per the organization structure participated in the study. These were, directors of Pfizer, GSK, and Astra Zeneca, General Managers of Bayer and Eli Lilly, and Regional Managers of Novartis and Roche. Whereas, 36 questionnaires were duly filled and returned by the business and supervising managers from production and marketing in each company. The reason behind sampling top managers and supervisors is that they are the subjects able to establish the firm's performance before and after patent expiry.

Data analysis involved obtaining both descriptive and inferential statistics using SPSS 22.0 to obtain central tendencies of the study outcomes presented in frequencies and mean scores and measures of dispersions in the form of variance and standard deviation. Correlation analysis was utilized to establish the relationship between the expiration of patents and the performance of innovator companies within the pharmaceutical industry in Kenya.

## Findings and Discussion

The study aimed to assess the effect of patent expiry on the performance of Innovator Multinational Pharmaceutical Corporations in Kenya by evaluating eight target companies. The study objectives first involved examining the effect of generic products and competitive market pressures after patent expiry concerning innovator multinational pharmaceutical companies' performance in Kenya. Second, to evaluate price changes and their effect on the performance of innovator multinational pharmaceutical companies after patent expiry. Finally, to explore the differences in sales volumes and profitability of innovator multinational pharmaceutical companies after patent expiry. A Likert scale ranged from strongly disagrees to strongly agree on the LOS through patent expiration for innovator companies. A value of 3.5 and above was taken to indicate agreement with the statement on generic production due to patent expiration and the change in competitive market pressures (Table 3).

Table 1 above represents data on price changes. Based on Table 1 analyis, the quantitative survey indicates agreement with customers opting for generic products due to lower prices at a mean of 4.11 and stiff competition arising due to patent expiration at a mean of 3.94. Furthermore, changes in the price of branded products off-patent were indicated not to occur with a mean of 3.17 and 3.29. This finding correlated to empirical data (6) that showed a lack of country-specific analysis. However, prior studies in the last decade on the generic dispensing ratio by Liberman and Roebuck (58) attributed changes to reducing gross pharmacy expenditure and savings. Competitive prices are evident with Crestor generics priced at 80–90% lower than the price of the original Crestor brand at $260 per month (59). This resulted in the original brand losing substantive market, declining sales and profitability as exemplified by 4.7. Pfizer's Lipitor generic production resulted in dropped prices with US patients seeing their monthly charges of the cholesterol-cutting drug from $30-$35 to around $5–$7 (60). The same was further experienced in the United States, where to maintain market share in the face of LOE, Sanofi's experience with Ambien CR and Pfizer's experience with Lipitor stand out (61). Looking at broader markets such as the United States, where regulation is put in place to protect innovator companies, the cost of medication is cited to be very high. Therefore, the ease of access to generic products in Kenya and the failure of innovator companies to patent their products within the Kenyan market favors generic penetration and result in highly competitive prices among generic products.

**Table 1:** Target population and sample frame.

	**Interview**		**Questionnaire survey**		
**Innovator company**	**Top managers**	**Sample size**	**Target population**	**Sample size**	**% of target popu** **lation**
Pfizer	1	1	30	4	13.3
GSK	1	1	44	6	13.6
Bayer	1	1	41	6	14.6
Sanofi Aventis	1	1	42	6	14.2
Novartis	1	1	34	5	14.7
Astra Zeneca	1	1	43	6	14.0
Eli Lilly	1	1	28	4	14.3
Roche	1	1	21	3	14.3
Total	8	8	283	40	

**Table 2:** Distribution of the respondents from the sample size.

	**Respondents interviewed**		**Questionnaire dully filled from the survey**		
	**Top managers**	**Sample size**	**Business managers**	**Sample size**	**% of respon** **dent**
Pfizer	Director	1	30	4	11.1
GSK	Director	1	44	5	13.8
Bayer	General Manager	1	41	5	13.8
Sanofi Aventis	Director	1	42	6	14.2
Novartis	Regional Director	1	34	4	11.1
Astra Zeneca	Director	1	43	5	13.8
Eli Lilly	General Manager	1	28	4	11.1
Roche	Regional Manager	1	21	3	8.3
Total	8	8	283	36	

**Table 3:** Changes in price descriptive statistics.

	***N***	**Min**	**Max**	**Mean**		**Std. deviation**	**Variance**
				**Statistic**	**Std. error**	**Statistic**	**Statistic**
Branded product price fall after patent expiry	36	1	4	2.60	0.137	0.812	0.659
No price change after patent expiry	36	1	5	3.17	0.156	0.923	0.852
Patent expiry affects prices of branded products	36	1	5	3.29	0.162	0.957	0.916
Customers opt for products with cheaper prices	36	2	5	4.11	0.158	0.932	0.869
Stiff price competition arises	36	1	5	3.94	0.169	0.998	0.997
Valid N	36						

The LOE immediately causes a steep decline in drug sales revenues, with sales units declining significantly when generic hit the market front. The study findings showed a positive correlation between patent expiration which leads to LOS for innovator companies leading to generic products getting into the market. For example, Lilly's LOE for Cymbalta drug resulted in more than 10 companies competing to manufacture generic versions of the drug (62). Furthermore, secondary data evaluated showed a substantial decline in revenues and profitability after patent expiry with a good example being Avodart, a drug manufactured by GSK and used in the management of benign hyperplasia, whose patent expired in November, 2015, resulting in a sharp drop in revenues from an average of $450 million in 2015 to about $80 million in 2016 (63). The change is a 5-fold drop in sales revenue, indicating a substantial decline in performance. DeRuiter and Holston (50) state that once a drug loses its copyright protections, low-priced generic products abruptly come into the market, siphoning as much as 90% of the sales.

The study explored the changes in sales volumes and profitability of innovator multinational pharmaceutical companies after patent expiry. This objective was crucial in evaluating the company's overall performance by looking into the sale volumes and profitability, which was supported by secondary data as provided in Table 4.

**Table 4:** Empirical data on sales units and volumes of Kenya 2019–2021.

	**Unit MAT/M09/2019 (absolute)**	**Unit MAT/M09/2020 (absolute)**	**Unit MAT/M09/2021 (absolute)**	**USDValue MAT/M09/2019 (absolute)**	**USDValue MAT/M09/2020 (absolute)**	**USDValue MAT/M09/2021 (absolute)**
Atorvastatin	48,399	45,060	53,837	983,909.64	891,321.36	1,097,678.78
Lipitor	48,399	45,060	53,837	983,909.64	891,321.36	1,097,678.78
Rosuvastatin	41,701	45,193	51,836	765,434.12	831,705.88	924,028.57
Crestor	41,701	45,193	51,836	765,434.12	831,705.88	924,028.57
Clopidogrel	4,794	5,067	4,367	143,767.31	165,187.51	152,964.21
Plavix	4,794	5,067	4,367	143,767.31	165,187.51	152,964.21
Valsartan	2,673	2,500	2,511	92,033.53	85,891.88	88,564.06
Diovan	2,673	2,500	2,511	92,033.53	85,891.88	88,564.06
Duloxetine	2,919	2,187	1,705	128,756.30	97,012.16	74,632.34
Cymbalta	2,919	2,187	1,705	128,756.30	97,012.16	74,632.34

*Source: (64)*.

Table 5 demonstrates changes on sales volume and profitability. The extent of price reductions following the entry of multiple generic products affects the sales volume and varies between products (65). Findings demonstrate a significant agreement with statements on the changes in sales volume and profitability changes after patent expiry. These findings are in tandem with (61) modeling of data in developed countries such as the United States. Patent expiration results in the declined financial performance of innovator companies with a mean response of 4.29, sales volume decrease, profit margins decline, and market share, as well as customer base change at mean values of 4.26, 3.91, and 3.86. Key in-depth findings indicated an overall reduction in the sales volume and profitability after the patent expiry of a given drug. A good example involved Diovan's patent expiry in 2013 and subsequent production of generics in the US market in 2015 resulted in a decline of sales from $3.5 billion to $1.7 billion (66).

**Table 5:** Sales volume and profitability descriptive statistics.

	***N***	**Min**	**Max**	**Mean**		**Std. deviation**	**Variance**
**Indicators**				**Statistic**	**Std. error**	**Statistic**	**Statistic**
Results in decreased financial performance	36	2	5	4.29	0.151	0.798	0.798
Decreased sales volume	36	2	5	4.26	0.180	1.138	1.138
Profit margins decline significantly	36	1	5	3.91	0.180	1.139	1.139
Slowed movement of branded pharmaceutical products	36	2	5	3.06	0.136	0.644	0.644
Market share and customer base change with patent expiry	36	2	5	3.86	0.160	0.891	0.891
Valid N	36						

Furthermore, interviews established that the Kenyan pharmaceutical industry has a smaller market share that makes innovator companies not invest heavily for fear of not recouping their revenues. However, there is a direct correlation between patent expiration and generic production as patented drugs must lose exclusive rights for generic companies to commence production. According to Chao et al. (67) in the global pharmaceutical market, the patent cliff has triggered essential decision-making processes for future policies to enhance the performance of innovator companies. The study established the need for innovator companies to aggressively engage the market dynamics to increase the market share and customer base.

Analysis of variance (ANOVA) is a statistical measure of comparing the differences between two or more means in a study. One-way ANOVA was utilized to establish the significance of the study outcome on patent expiry and indicates a positive coefficient. These findings are summarized in Table 6 as follows.

**Table 6:** Analysis of variance.

**ANOVA**				**Sum of squares**	**df**	**Mean square**	**F**	**Sig**.
Generic products	Between groups	(Combined)		11.440	6	2.860	2.471	0.066
		Linear term	Unweighted	1.478	1	1.478	1.276	0.268
			Weighted	0.879	1	0.879	0.759	0.390
			Deviation	10.561	3	3.520	3.041	0.044
	Within groups			34.731	30	1.158		
	Total			46.171	36			
Price change	Between groups	(Combined)		5.948	4	1.487	2.105	0.105
		Linear term	Unweighted	0.412	1	0.412	1.217	0.451
			Weighted	0.860	1	0.860	0.583	0.279
			Deviation	5.088	3	1.696	2.401	0.087
	Within groups			21.195	30	0.707		
	Total			27.143	36			
Sales volumes and profitability	Between groups	(Combined)		4.310	4	1.077	0.939	0.455
		Linear term	Unweighted	1.859	1	1.859	0.620	0.213
			Weighted	0.891	1	0.891	0.647	0.209
			Deviation	2.419	3	0.806	0.702	0.558
	Within groups			34.433	30	1.148		
	Total			38.743	36			

As an aspect of the independent variable patent expiration, generic products resulted in a weighted *F*-value of 0.75; price change 0583; with sales volume and profitability 0.647. The *F*-values are significantly smaller showing the variance in the means does not affect the relationships between the various aspects in the study variables. Thus, the relationship between patent expiry and generic production is significant in affecting the performance of innovator companies. Changes in the financial position within Novartis's Diovan points to profits declining by 16.6% in 2012 linked to patent expiry (68). Stagnant revenues in the company failed to compensate for the weaker sales realized by the Diovan affecting the company's overall performance. Increased pressure on the already existing market for the product is associated with the emergence of generic products that hit the market at much lower prices. This effect is being considered by health technology assessment agencies in New Zealand, Norway, and France who already require drug manufacturers to consider the impact of generic entry on future prices (65). The findings are supported by secondary data from the systematic review of target companies' performance after patent expiration. Pfizer's Lipitor was a bestselling drug in the 1990s and early 2000s, but the LOS in 2011 saw a significant reduction in sales with 19% decline recorded in the first quarter of 2012. The most recent data from Kenya presented in the table for the financial period 2019–2021 indicates a continuous decline. The strategic perspective of this trend can be linked to the theoretical view of competitive strategies that require managerial decisions based on the entrant of competitors. Consequently, to understand these attributes and their relationship to the brand's potential to retain share, informed brand management strategies can better determine what strategies will be most effective once generics enter the market for the multinational innovator firms.

The projections are based on the increased competition that arises with generic brands flooding the market shrinking the customer base for original brands. Furthermore, Pfizer's Lyrica, an anticonvulsant drug, went off-patent in December 2018, and projections are showing a substantial decline in sales volumes in 2019 from $5 billion in 2018 to $3 billion (69). Nevertheless, the drug will only be utilized for shingles and diabetic peripheral neuropathy, thereby limiting the original Lyrica's potential.

GSK's Avodart had its patent expiring in November 2015 and resulted in a revenue drop from an average of $450 million in 2015 to about $80 million in 2016 (63). Results in declined performance for GlaxoSmithKline, an international innovator company, are attributed to patent expiry. Eli Lilly in 2017 saw a 7.2% decline in sales after Strattera went off-patent (70). Additionally, Cymbalta's patent expiration in 2013 resulted in the sales decline of the drugs by 25.41% in the fourth quarter. Essentially, there is a direct correlation between patent expiry and production of generics, price increase, and declining sales volume performance. According to Mullin (5), the LOS in the innovator pharmaceutical companies exposes them to competitive pressures and diminishes the market base for improved performance. Furthermore, Ngamau (71) asserted that patent expiration revealed branded products to increased competitive prices as generic products trade at reasonably low prices; thus, they become the most option for many clients, especially in Kenya.

### Theoretical Implications

The entry of generic products into the market after the loss of patent or innovations of emerging products is implied in Porter Competitive forces. The market forces that include customers, suppliers, potential entrants, and substitute products are considered as managerial imperatives for strategic decisions. The strategic imperative for a perfectly competitive industry is challenged when entry is made easy due to loss of exclusivity. Patent expiration opens up the innovator pharmaceutical companies into the realities of market competitive pressures where generics are imminent and price control is not within the advantage of the company. Particular organizations establish a system of operation that is independent of the concrete substance of elements (72). Thus, the same concepts and principles of an organization underlie different disciplines of operation that provide a basis for the union to work as a single entity. The system concept incorporates settings of an organization, the inputs and output, process, state, goals, hierarchy, and information in the sector, hence the theoretical perspectives are implied.

### Suggestions for Further Areas of Research

The Kenyan pharmaceutical industry that is of LMIC is small and emerging with great growth potential. However, it is dominated by generic products due to affordability and ease of access. There exist pockets of students' academic research in various areas of strategic management. There is a need for further studies to assess the market regulation and challenges affecting the uptake of branded products aside from the price factor. The specific market in-depth study should be carried out concerning the lack of patented drugs in the Kenyan market and its impact on innovator pharmaceutical companies.

### Practical Implications and Policy Recommendations

The Pharmaceutical Industry: Research findings provide a guide for Multinational Pharmaceutical Corporations to attain strategies for sustainable growth and performance in the industry. The research's finding will be crucial to future innovator companies as a benchmark to leverage during entry to the market, as well as those already existing in the market on how to sustain growth after LOEs.

Pharmaceutical Companies: The study established that Multinational Pharmaceutical Corporations ought to come up with strategies to overcome challenges affecting their operations after patent expiry. Furthermore, establish effective strategies that can be useful to create sustainable competitive advantage after their products' patent expires.

Policy Makers: The study provides insights into the need to come up with strategies or measures to shield innovator companies from disadvantaged competition by generic products after extensive R&D on a product. Innovator companies should acquire capacity and higher stakes on the returns acquired from generic products of their patented innovations even after expiry.

## Conclusions

Findings indicate a direct correlation between patent expiration and generic production with the LOS, leading to massive production of generic products by approved companies. These findings from Low Middle Income Country confirms (61) output on measuring and modeling healthcare cost. The LOE leads to increased manufacture of cheaper generic versions and immediate flooding in the market, constricting the market base for original branded products. Increased market competitive pressures experienced with the Loss of rights for exclusivity for the products resulting in sales decline and subsequent drop in profitability.

The LOE results in competitive pricing strategies as companies assess market dynamics. However, the study indicates that generic products dominate Kenya, a Low Middle Income country, due to their lower prices, which significantly reduces the market share for branded products. Thus, innovator multinational pharmaceutical companies face the challenge of tapping this market due to the availability of cheaper options at the expense of the expensive branded drugs in the low middle-income level. However, this is not the case for high-income countries as demonstrated by Aitken et al. (61) in their modeling on market response.

On sales volume and productivity, this study concludes that increased competition by generic products, which shrink the market share and customer base for the branded products by competing effectively at low prices, is a major contributing factor to the low performance of multinational innovator companies. Individual drugs sales revenues showed a significant decline in sales volumes and revenues off-patent. Innovator companies are thus, exposed to a competitive market that eventually shrinks their customer base, which results in reduced profitability.

## Data Availability Statement

The raw data supporting the conclusions of this article will be made available by the authors, without undue reservation.

## Ethics Statement

Ethical review and approval was not required for the study on human participants, in accordance with the local legislation and institutional requirements.

## Author Contributions

FK conceptualized the study, collected the data, and conducted a preliminary analysis. JO provided guidance on the conceptualization of the study as the supervisor and developed the manuscript. Both authors contributed to the article and approved the submitted version.

## Conflict of Interest

FK was employed by the company Getz Pharma (Kenya). The remaining author declares that the research was conducted in the absence of any commercial or financial relationships that could be construed as a potential conflict of interest.

## Publisher's Note

All claims expressed in this article are solely those of the authors and do not necessarily represent those of their affiliated organizations, or those of the publisher, the editors and the reviewers. Any product that may be evaluated in this article, or claim that may be made by its manufacturer, is not guaranteed or endorsed by the publisher.
